# Extended versus conventional letrozole regimen in patients with polycystic ovary syndrome undergoing their first ovulation induction cycle: a prospective randomized controlled trial

**DOI:** 10.1093/hropen/hoae046

**Published:** 2024-07-18

**Authors:** Xiuxian Zhu, Jingwen Lang, Qiaoling Wang, Yonglun Fu

**Affiliations:** Department of Assisted Reproductive Medicine, Shanghai First Maternity and Infant Hospital, School of Medicine, Tongji University, Shanghai, PR China; Shanghai Key Laboratory of Maternal Fetal Medicine, Shanghai Institute of Maternal-Fetal Medicine and Gynecologic Oncology, Shanghai First Maternity and Infant Hospital, School of Medicine, Tongji University, Shanghai, PR China; Department of Assisted Reproductive Medicine, Shanghai First Maternity and Infant Hospital, School of Medicine, Tongji University, Shanghai, PR China; Shanghai Key Laboratory of Maternal Fetal Medicine, Shanghai Institute of Maternal-Fetal Medicine and Gynecologic Oncology, Shanghai First Maternity and Infant Hospital, School of Medicine, Tongji University, Shanghai, PR China; Department of Assisted Reproductive Medicine, Shanghai First Maternity and Infant Hospital, School of Medicine, Tongji University, Shanghai, PR China; Shanghai Key Laboratory of Maternal Fetal Medicine, Shanghai Institute of Maternal-Fetal Medicine and Gynecologic Oncology, Shanghai First Maternity and Infant Hospital, School of Medicine, Tongji University, Shanghai, PR China; Department of Assisted Reproductive Medicine, Shanghai First Maternity and Infant Hospital, School of Medicine, Tongji University, Shanghai, PR China; Shanghai Key Laboratory of Maternal Fetal Medicine, Shanghai Institute of Maternal-Fetal Medicine and Gynecologic Oncology, Shanghai First Maternity and Infant Hospital, School of Medicine, Tongji University, Shanghai, PR China

**Keywords:** ovulation induction, polycystic ovary syndrome, letrozole, extended letrozole regimen, ovarian hyperstimulation syndrome

## Abstract

**STUDY QUESTION:**

Can an extended letrozole (LE) regimen result in a higher ovulatory rate than a conventional regimen in patients with polycystic ovary syndrome (PCOS) undergoing their first ovulation induction cycle?

**SUMMARY ANSWER:**

There was no statistical difference in ovulation rate between patients with PCOS using the extended LE regimen and those using the conventional LE regimen.

**WHAT IS KNOWN ALREADY:**

LE has become the first-line agent for ovulation induction. However, there is still a proportion of non-responsive cycles in patients with PCOS undergoing ovulation induction therapy with LE alone, and the extended LE regimen has been demonstrated to be a feasible method for inducing ovulation in these non-responders. Nevertheless, whether the extended regimen could be applied to all patients with PCOS as a first choice for the induction of ovulation remains to be explored.

**STUDY DESIGN, SIZE, DURATION:**

This was a prospective randomized controlled trial that included 148 female patients with PCOS who underwent their first ovulation induction cycle with LE from January 2021 to October 2022.

**PARTICIPANTS/MATERIALS, SETTING, METHODS:**

Participants were randomly assigned to receive an extended (5 mg LE daily for 7 days) or conventional regimen (5 mg LE daily for 5 days) for one treatment cycle. The ovulation rate was the primary outcome. Secondary outcomes included the clinical pregnancy rate, the number of preovulatory follicles, and the rate of multiple pregnancies.

**MAIN RESULTS AND THE ROLE OF CHANCE:**

The ovulation rate among patients receiving an extended LE regimen was slightly higher than the rate with a conventional LE regimen, but the difference did not reach statistical significance in either the intention-to-treat analysis (90.54% [67/74] vs 79.73% [59/74], *P* = 0.065; relative risk [95% CI]: 0.881 [0.768–1.009]) or the per-protocol analysis (90.54% [67/74] vs 84.29% [59/70], *P* = 0.257; relative risk [95% CI]: 0.931 [0.821–1.055]). The number of preovulatory follicles was nearly identical in the two groups (1.39 ± 0.62 vs 1.37 ± 0.59, *P* = 0.956), and no cases of ovarian hyperstimulation syndrome were observed. With regards to the endometrial parameters, the mean endometrium thickness was slightly thicker with the conventional LE regimen compared to that with the extended LE regimen, though with no statistical difference (9.27 ± 1.72 mm vs 9.57 ± 2.28 mm, *P* = 0.792). In the per-protocol analysis, the rates of clinical pregnancy (20.27% [15/74] vs 14.29% [10/70], *P* = 0.343; relative risk [95% CI]: 0.705 [0.34–1.463]) and live birth (13.51% [10/74] vs 11.43% [8/70], *P* = 0.705; relative risk [95% CI]: 0.846 [0.354–2.019]) did not differ significantly between treatment groups. Moreover, all conceptions were singletons without neonatal defects.

**LIMITATIONS, REASONS FOR CAUTION:**

The major concerns regarding this study are its single-center and open-label nature. Additionally, the limited number of lean patients with PCOS with a mean body mass index of 23–25 kg/m^2^ enrolled in our trial also restricted the generalizability of our findings.

**WIDER IMPLICATION OF THE FINDINGS:**

A change from the standard strategy of ovulation induction in patients with PCOS is not advisable, because a statistically superior effect of the extended LE regimen over a conventional regimen was not detected. The extended LE regimen could be applied with caution in a specific population who failed to respond to a conventional regimen rather than all the patients with PCOS during ovulation induction. Additional prospective trials with larger sample sizes and different PCOS subgroups are needed to assess the ovulatory effects of various LE treatment durations.

**STUDY FUNDING/COMPETING INTEREST(S):**

This study was funded by the Shanghai First Maternity and Infant Hospital, affiliated with Tongji University School of Medicine (grant numbers: 2023B03 to Y.F., 2023B18 to X.Z., and 2020RC02 to Y.F.). The authors report no conflicts of interest.

**TRIAL REGISTRATION NUMBER:**

Chinese Clinical Trial Registry (ChiCTR2100042082).

**TRIAL REGISTRATION DATE:**

13 January 2021.

**DATE OF FIRST PATIENT’S ENROLMENT:**

21 January 2021.

WHAT DOES THIS MEAN FOR PATIENTS? Letrozole has become the most commonly prescribed oral ovulation induction agent for subfertile women who do not ovulate naturally, particularly those with polycystic ovary syndrome (PCOS). However, there is still a proportion of patients with PCOS who fail to respond to conventional letrozole therapy. The extended letrozole regimen has been demonstrated to be a feasible method for inducing ovulation in these ‘non-responders’. Nevertheless, whether this may be applied to all patients with PCOS as the first choice for the induction of ovulation remains to be explored. Thus, we conducted this prospective randomized controlled study to assess whether an extended letrozole regimen is superior to the conventional letrozole regimen in patients with PCOS when undergoing their first ovulation induction cycle. Our data showed that the rate of ovulation among patients receiving an extended letrozole regimen was slightly higher than the rate with a conventional letrozole regimen group, but the difference did not reach statistical significance. In consideration of the potential risk of multi-follicular development with the extended letrozole regimen, which would require more monitoring and higher costs to avoid multiple pregnancies, we conclude that it is not necessary to replace the conventional regimen with an extended letrozole regimen in patients with PCOS during their first ovulation induction cycle.

## Introduction

Ovulatory dysfunction is a major cause of infertility in patients with polycystic ovary syndrome (PCOS) (Balen *et al.*, 2016; Teede *et al.*, 2023), and letrozole (LE) has become the most commonly prescribed oral ovulation induction agent (Wang *et al.*, 2019; Pundir *et al.*, 2021; Tsiami *et al.*, 2021; Franik *et al.*, 2022; Liu *et al.*, 2023). However, there is still a proportion of non-responsive cycles in patients with PCOS undergoing ovulation induction therapy with LE alone (Ramezanzadeh *et al.*, 2011; Legro *et al.*, 2014; Wu *et al.*, 2016; Amer *et al.*, 2017; Mejia *et al.*, 2019; Shi *et al.*, 2022; Dai *et al.*, 2023; Sharma *et al.*, 2023). Exogenous gonadotropin is the most commonly used drug for these non-responders with an increased risk of multiple-follicular development, subsequent cycle cancellation, severe ovarian hyperstimulation syndrome (OHSS), or multiple pregnancies (Weiss *et al.*, 2019; Chen *et al.*, 2023; Dai *et al.*, 2023; Xia *et al.*, 2023). Therefore, it is urgent to explore other options for inducing ovulation in those non-responsive patients.

Our team first proposed the method of extending the LE treatment duration step by step to induce follicle growth in patients with PCOS who could not achieve ovulation with the conventional 5-day LE regimen (Zhu and Fu, 2023), referred to as ‘LE resistance’ (Guo *et al.*, 2023; Neblett *et al.*, 2023). Our data showed that 48 out of 69 patients with LE resistance (69.57%) achieved ovulation with 7 days of treatment of LE 5 mg per day, and a further 16 patients (23.19%) ovulated after receiving another 10 days of treatment (Zhu and Fu, 2023). Since the ovulatory effect of the extended LE regimen was compelling, it was worth asking whether it could be applied to all patients with PCOS as a first choice for inducing ovulation. Thus, we designed this prospective randomized controlled study to assess whether an extended LE regimen is superior to the conventional LE regimen in patients with PCOS undergoing their first ovulation induction cycle.

## Materials and methods

### Ethical approval

The study protocol was approved by the hospital’s Institutional Review Board. The trial was registered with the Chinese Clinical Trial Registry (ChiCTR2100042082). Written informed consent was obtained from every participant before randomization.

### Study design and participants

This prospective randomized controlled study was conducted at the Shanghai First Maternity and Infant Hospital from January 2021 to October 2022.

Outpatients with PCOS who underwent their first ovulation induction cycle were screened. The inclusion criteria were as follows: (i) a diagnosis of PCOS based on a modified form of the Rotterdam criteria (Rotterdam ESHRE/ASRM-Sponsored PCOS Consensus Workshop Group, 2004): chronic anovulation or oligomenorrhea, clinical or biochemical signs of hyperandrogenism, and polycystic ovaries on ultrasonography; (ii) aged 20–35 years; and (iii) normal semen analysis of their male partners.

Patients with a previous history of ovulation induction failures or other diseases such as active thyroid disease, congenital adrenal hyperplasia, hyperprolactinemia, androgen-secreting tumors, Cushing’s syndrome, and clinically significant systemic diseases were excluded. In addition, those who had received drugs that interfered with hormone levels in the previous three months were not enrolled in our study.

### Interventions

Participants were randomly assigned to receive an extended or conventional LE regimen for one treatment cycle. Specifically, LE 5 mg (Letrozole tablets; Yimeishu^®^, Zhejiang Hisun Pharmaceutical Co., Ltd, Taizhou, China) was prescribed orally daily for seven consecutive days in the study group and for five consecutive days in the control group, starting on days 2–4 of the menstrual period or progesterone (P)-induced bleeding. To observe the ovarian response in as much detail as possible, an ultrasound was performed every 2–4 days after the last dose of LE to record the number/size of follicles and endometrial thickness; meanwhile, serum hormone levels were determined with a chemiluminescent method (Abbott Biologicals B.V., Weesp, The Netherlands). The lower limits of sensitivity were as follows: FSH 0.06 IU/l, luteinizing hormone (LH) 0.09 IU/l, estradiol (E_2_) 10 pg/ml, and P 0.1 ng/ml. The E_2_ values were recorded as 5000 pg/ml if it surpassed 5000 pg/ml.

Ovulation was diagnosed by the disappearance of a follicle greater than 14 mm or a serum P level >3 ng/ml followed by pregnancy or menses. If no follicles with a diameter >10 mm were detected, concomitant with E_2_ <70 pg/ml and *P* < 1.0 ng/ml 14 days after the last dose of LE, dydrogesterone (Duphaston; Abbott Biologicals B.V.) was delivered to induce bleeding.

Couples were advised to engage in regular intercourse every 2–4 days and timed intercourse if an LH surge was detected. Serum hCG levels were tested 2 weeks after ovulation to diagnose conception and an ultrasound was performed 4 weeks to diagnose a clinical pregnancy. Pregnancy and neonatal outcomes were tracked through a review of maternal and infant medical records. A nurse supervised and checked whether participants had complied well with their prescriptions by asking and checking the surplus LE tablets.

### Outcome variables

The primary outcome was ovulation rate; secondary outcomes included the clinical pregnancy rate, number of preovulatory follicles (with a diameter larger than 14 mm), and rate of multiple pregnancies (the presence of two or more gestational sacs in the uterine cavity). Other outcomes included the largest follicle diameter, endometrial thickness, time-to-ovulation (the number of days from the first dose of LE to ovulation), mono-follicular rate (cycles with one follicle ≥14 mm per ovulatory cycle), and rates of OHSS, biochemical pregnancy, early miscarriage (loss of pregnancy before 12 weeks of gestation), and live birth (defined as a live baby born after 28 weeks of gestation). Both ectopic pregnancies and the presence of a gestational sac in the uterus, as determined using ultrasonography, were considered clinical pregnancies.

### Sample size and randomization

To demonstrate a clinically meaningful difference of 20% between the previously reported ovulation rate of the conventional (∼75%) and extended LE regimen with a two-sided significance level of 0.05 and power of 85%, a sample size of 132 participants (66 per arm) was required (PASS 15.0.5). The sample size was increased to 74 participants per arm to allow for ∼10% dropouts.

The enrolled participants were allocated in a ratio of 1:1 to receive treatment with either the extended or the conventional LE regimen according to a randomization list generated by the trial statistician using Statistical Analysis System (SAS) 9.4. The enrolment order and the treatment code (A or B) were sealed in an opaque envelope with random numbers. Participants and physicians were not blinded to the group assignment. However, the sonographers were blinded to the group assignments in the trial.

### Statistical analyses

The intention-to-treat (ITT) analysis included all randomized participants regardless of whether they were lost to follow-up, whereas the per-protocol (PP) analysis included those who completed the allocated treatment and follow-up. Participants who were lost to follow-up were assumed neither to ovulate nor be pregnant in the ITT analysis.

Categorical variables were assessed using the chi-squared test or Fisher’s exact test. Student’s *t*-test was used to assess continuous variables in each normally or near-normally distributed group, and the Mann–Whitney *U* test was used to assess continuous variables with a non-normal distribution. Statistical analyses were performed using SPSS 20 for Windows (release 6.0; IBM Corporation, Armonk, NY, USA). All *P*-values were two-sided, and a *P*-value of <0.05 was considered statistically significant.

## Results

### Characteristics of the participants

**Figure 1.**
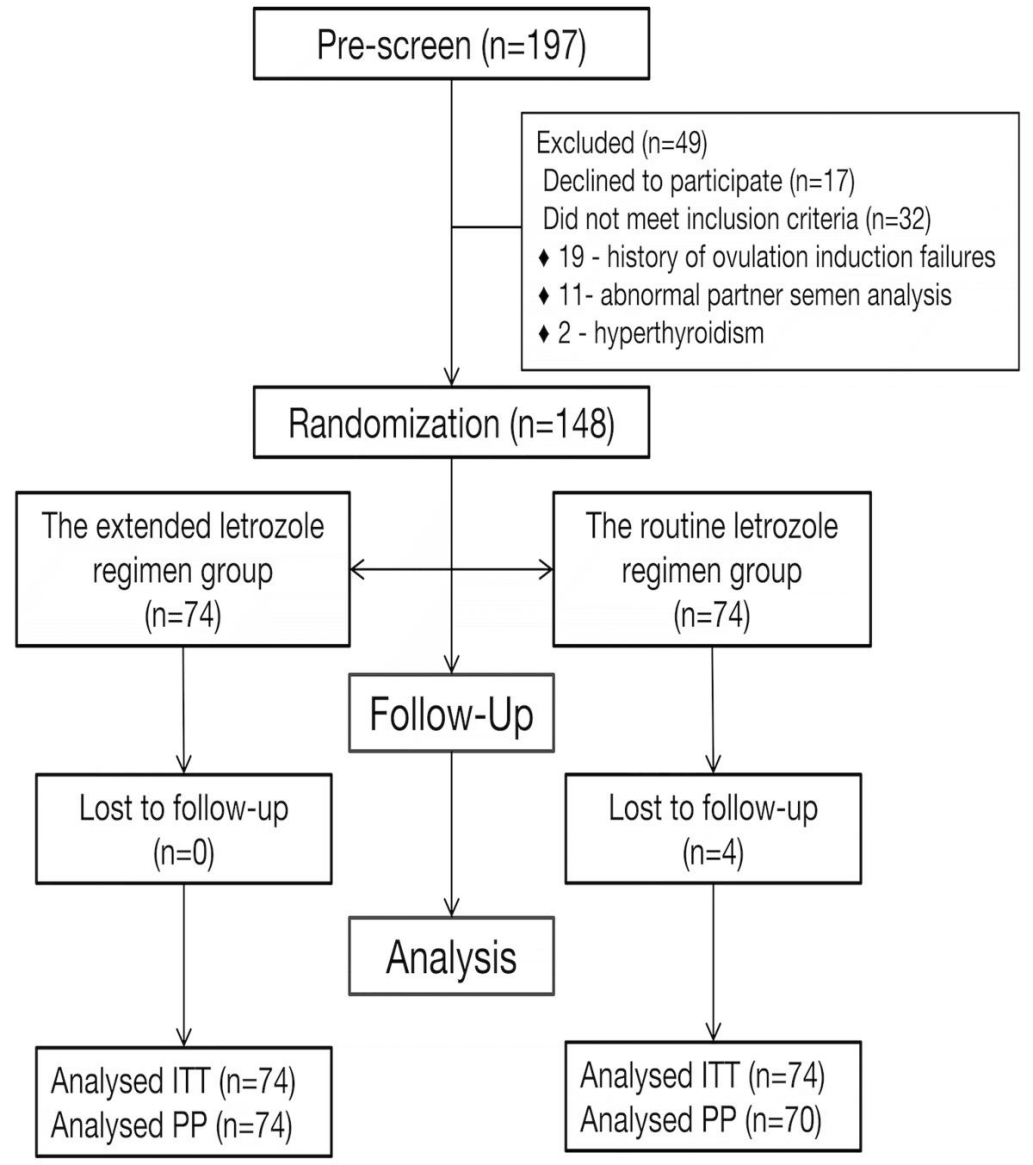
**Flowchart of the study.** ITT, intention-to-treat; PP, per-protocol.

Figure 1 presents a flowchart of the trial. A total of 197 patients with PCOS were screened and 148 were enrolled in this study. Four patients in the conventional LE regimen group were lost to follow-up after their first visit. The remaining 144 completed the allocated treatments. As shown in Table 1, the mean maternal age in the extended LE regimen group was higher than that in the conventional LE regimen group. However, the difference was not clinically significant. Other baseline characteristics of the two groups were similar.

**Table 1.** Baseline characteristics of participants.

Characteristic	The extended letrozole regimen	The conventional letrozole regimen	*P*-values
	(n = 74)	(n = 74)	
Maternal age (years)	29.68 ± 3.44	28.40 ± 3.15	0.023*
Infertility duration (years)	2.1 ± 1.36	1.93 ± 1.27	0.426*
AMH (ng/ml)	10.21 ± 4.87	9.72 ± 4.32	0.740*
BMI (kg/m^2^)	24.04 ± 4.3	23.5 ± 3.6	0.513*
Number of patients in each BMI group, n (%)			
<18.5 kg/m^2^	5 (6.76%)	4 (5.41%)	0.849^†^
18.5–25 kg/m^2^	39 (52.7%)	45 (60.81%)	
25–30 kg/m^2^	25 (33.78%)	21 (28.38%)	
≥30 kg/m^2^	5 (6.76%)	4 (5.41%)	
Type of infertility, n (%)			
Primary infertility	51 (68.92%)	56 (75.68%)	0.358^‡^
Secondary infertility	23 (31.08%)	18 (24.32%)	
Menstrual dysfunction, n (%)			
Oligomenorrhea	65 (87.84%)	67 (90.54%)	0.597^‡^
Amenorrhea	9 (12.16%)	7 (9.46%)	
PCOS diagnosis, n (%)			
Polycystic ovaries	74 (100%)	74 (100%)	0.756^†^
Hyperandrogenism (clinical or laboratory)	3 (4.05%)	1 (1.35%)	
Menstrual dysfunction	74 (100%)	74 (100%)	
Fallopian tube patency, n (%)			
One patent tube	6 (8.11%)	5 (6.76%)	0.648^‡^
Bilateral patent tube	18 (24.32%)	23 (31.08%)	
No test records	50 (67.57%)	46 (62.16%)	

Data are presented as n, mean ± SD or n (%).

* Mann–Whitney *U* test (for continuous variables).

† Fisher’s exact test (for categorical variables).

‡ Pearson chi-square (for categorical variables).

AMH, anti-Müllerian hormone; BMI, body mass index; PCOS, polycystic ovary syndrome.

### Cycle characteristics

As shown in Table 2, the ovulation rate among patients receiving an extended LE regimen was slightly higher than the rate with a conventional LE regimen, but the difference did not reach a statistical significance in either the ITT analysis (90.54% [67/74] vs 79.73% [59/74], *P* = 0.065; relative risk [95% CI]: 0.881 [0.768–1.009]) or the PP analysis (90.54% [67/74] vs 84.29% [59/70], *P* = 0.257; relative risk [95% CI]: 0.931 [0.821–1.055]).

**Table 2.** Reproductive outcomes.

Measured parameters	The extended letrozole regimen	The conventional letrozole regimen	*RR (95% CI)*	*P*-values
**Intention-to-treat analysis**				
Ovulation rate	67/74 (90.54%)	59/74 (79.73%)	0.881 (0.768–1.009)	0.065^‡^
Clinical pregnancy rate	15/74 (20.27%)	10/74 (13.51%)	0.667 (0.321–1.387)	0.273^‡^
Multiple pregnancy rate	0/15 (0%)	0/10 (0%)	—	—
Live birth rate	10/74 (13.51%)	8/74 (10.81%)	0.82 (0.34–1.973)	0.656^‡^
**Per-protocol analysis**				
Ovulation rate	67/74 (90.54%)	59/70 (84.29%)	0.931 (0.821–1.055)	0.257^‡^
Biochemical pregnancy rate	17/74 (22.97 %)	12/70 (17.14%)	0.746 (0.385–1.448)	0.383^‡^
Clinical pregnancy rate	15/74 (20.27%)	10/70 (14.29%)	0.705 (0.34–1.463)	0.343^‡^
Multiple pregnancy rate	0/15 (0%)	0/10 (0%)	—	—
Ectopic pregnancy rate	1/15 (6.67 %)	0/10 (0%)	—	—
Early miscarriage rate	4/15 (26.67%)	2/10 (20%)	0.75 (0.168–3.351)	1.000^†^
Live birth rate	10/74 (13.51%)	8/70 (11.43%)	0.846 (0.354–2.019)	0.705^‡^

Data are presented as n, mean ± SD or n (%).

† Fisher’s exact test (for categorical variables).

‡ Pearson chi-square (for categorical variables).

CI, confidence interval; RR, relative risk.

**Figure 2.**
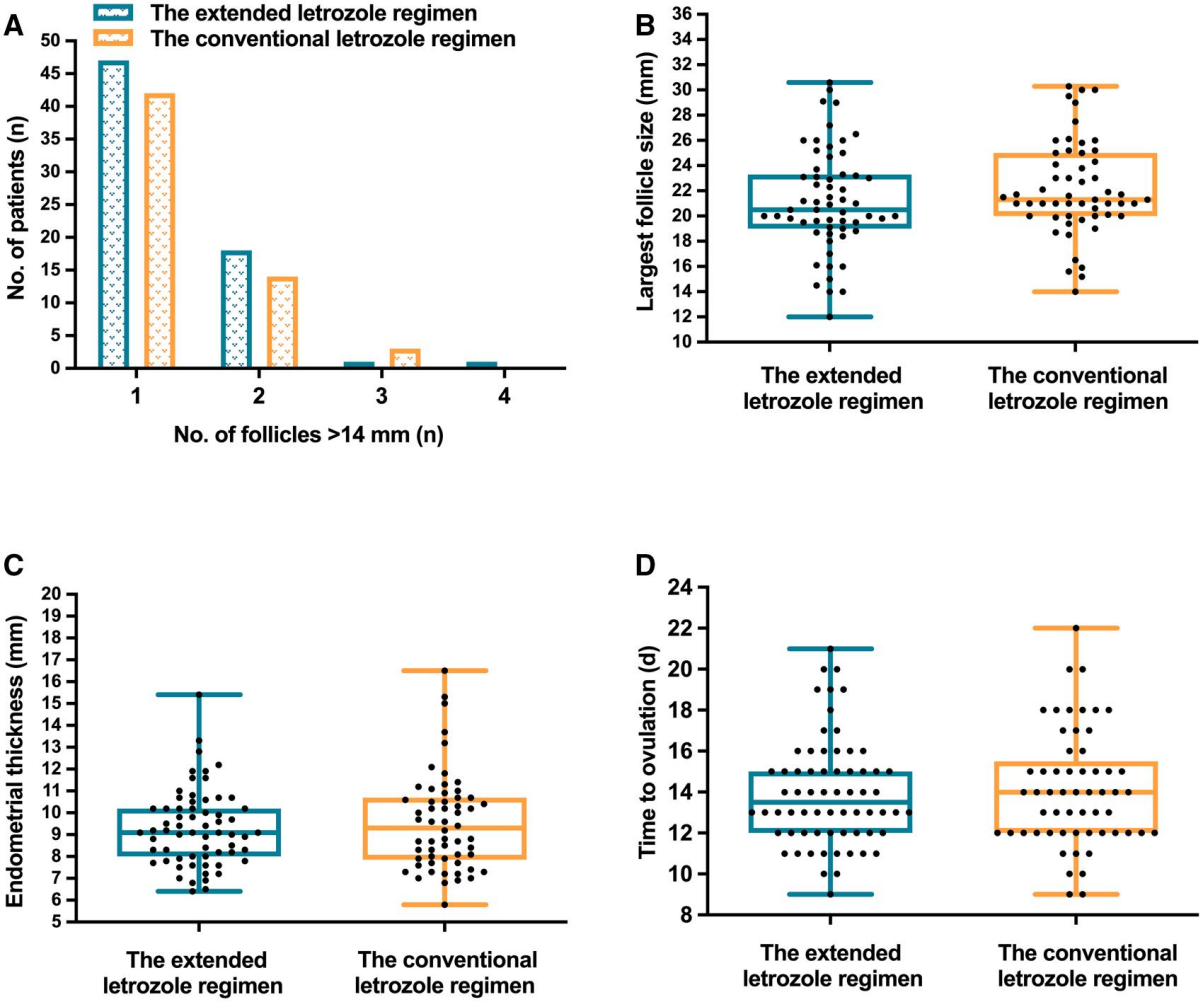
**Cycle characteristics.** (**A**) The distribution of women in the two groups with follicle diameter >14 mm; (**B**) the largest follicle diameter; (**C**) endometrial thickness; and (**D**) the time of ovulation in ovulatory cycles. Time-to-ovulation refers to the number of days from the first dose of letrozole administration to ovulation. The blue boxes represent the women who underwent the extended letrozole regimen, and the orange boxes represent those who underwent the conventional letrozole regimen.

Among patients who ovulated, the mono-follicular and bifollicular rates were comparable between the two groups (Fig. 2A, Table 3). One woman in the study group and three patients in the control group yielded three dominant follicles, while four preovulatory follicles were found in one of the participants receiving the extended LE regimen (Table 3). No significant differences were found between groups when it came to the number of preovulatory follicles, the average largest follicle size, time-to-ovulation, or endometrial thickness (Table 3). The largest follicle diameter documented before ovulation was 30.6 mm among the extended LE regimen recipients and 30.3 mm among the conventional LE regimen recipients (Fig. 2B). All patients ovulated spontaneously without triggers, and no cases of OHSS were reported in either group (Table 3). With regards to the endometrial parameters (Fig. 2C, Table 3), the mean endometrium thickness was slightly thicker with the conventional LE regimen compared to the extended LE regimen, though with no statistical difference (9.27 ± 1.72 mm vs 9.57 ± 2.28 mm, *P* = 0.792). In addition, only four patients in the extended LE regimen group and three in the conventional LE regimen group showed endometrial thickness <7 mm (Fig. 2C). Time-to-ovulation ranged from 9 to 21 days among patients who received the extended LE regimen, similar to that among patients who received the conventional LE regimen (9–22 days) (Fig. 2D).

**Table 3.** Cycle characteristics in ovulatory cycles.

Measured parameters	The extended letrozole regimen	The conventional letrozole regimen	*P*-values
	(n = 67)	(n = 59)	
Number of preovulatory follicles	1.39 ± 0.62	1.37 ± 0.59	0.956*
Largest follicle size (mm)	21.35 ± 3.88	22.18 ± 3.72	0.251*
Time-to-ovulation (days)	13.97 ± 2.61	14.11 ± 2.81	0.84*
Endometrial thickness (mm)	9.27 ± 1.72	9.57 ± 2.28	0.792*
Mono-follicular rate, % (n)	47/67 (70.15%)	42/59 (71.19%)	0.898^‡^
Bifollicular rate, % (n)	18/67 (26.87%)	14/59 (23.73%)	0.686^‡^
Cycles of spontaneous ovulation	67	59	—
OHSS cases	0	0	—

Data are presented as n, mean ± SD or n (%).

* Mann–Whitney *U* test (for continuous variables).

‡ Pearson chi-square (for categorical variables).

OHSS, ovarian hyperstimulation syndrome.

### Hormone profiles during treatment

**Figure 3.**
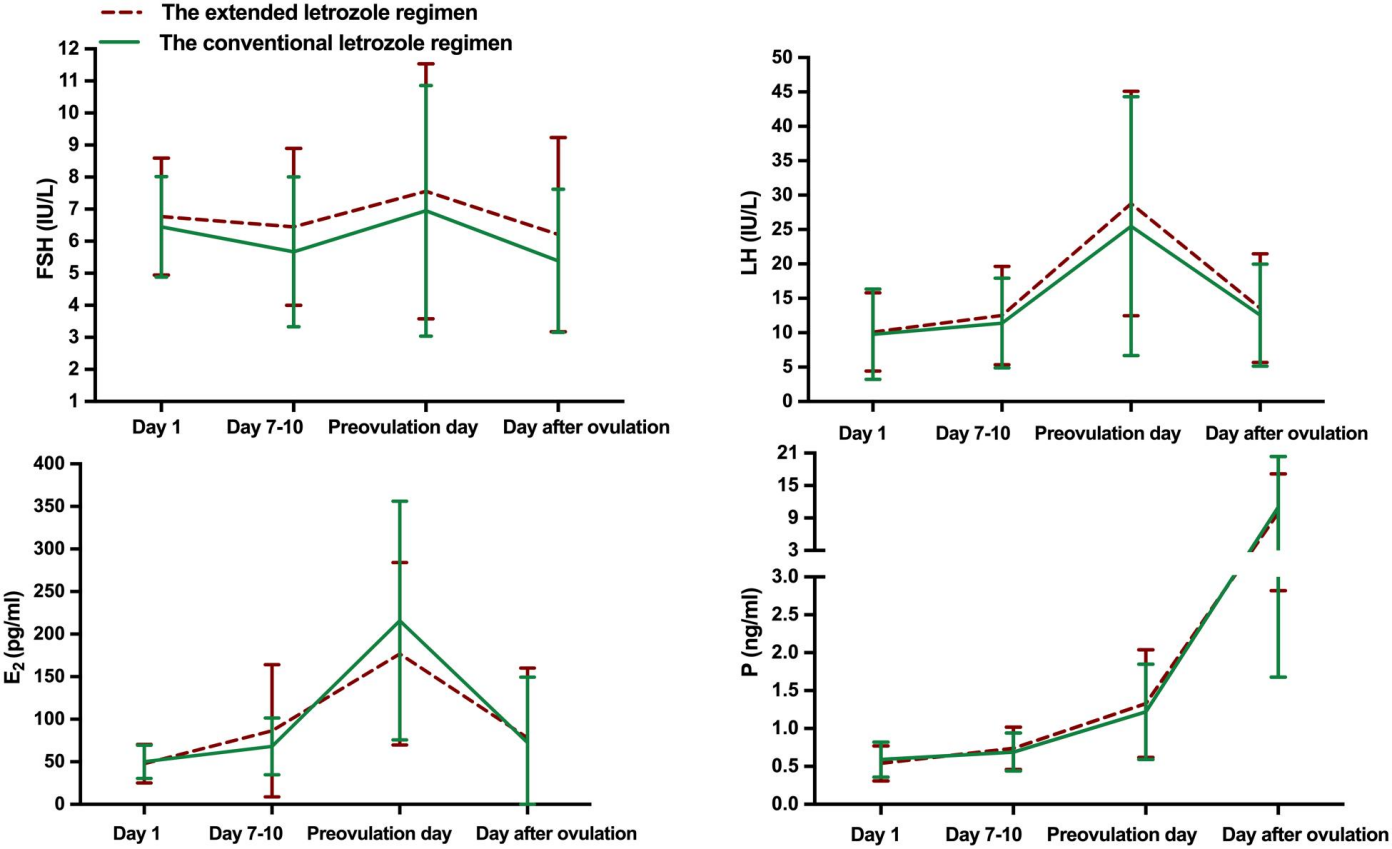
**Hormone profile during treatment.** Serum hormone levels during ovarian stimulation in ovulatory cycles in the two letrozole regimens. The red lines represent the extended letrozole regimen group, and the green lines represent the conventional letrozole regimen group. The day of treatment initiation was recorded as Day 1 of each regimen. *P < 0.05 at the time point. FSH, follicle-stimulating hormone; LH, luteinizing hormone; E_2_, estradiol; P, progesterone.

Figure 3 depicts the dynamic hormone profiles of the ovulatory cycles in all participants. Day 1 represents the day on which LE treatment was initiated. The mean FSH levels were slightly higher in the extended LE regimen than in the conventional LE regimen group throughout the ovulation induction cycle; however, the differences were not statistically significant. No between-group differences were found in the average serum LH levels on Days 7–10 and on the pre-ovulation day. The average E_2_ levels on the pre-ovulation day were higher in the conventional LE regimen group than in the extended LE regimen group, although the difference was not statistically significant. The serum *P* levels of the two groups were similar during treatment.

### Reproductive outcomes

In the PP analysis, the rates of clinical pregnancy (20.27% [15/74] vs 14.29% [10/70], *P* = 0.343; relative risk [95% CI]: 0.705 [0.34–1.463]) and live birth (13.51% [10/74] vs 11.43% [8/70], *P* = 0.705; relative risk [95% CI]: 0.846 [0.354–2.019]) did not differ significantly between the treatment groups (Table 2), and similar outcomes were noted in the ITT analysis. All pregnant patients in our study were diagnosed as having singleton pregnancies. Four miscarriages occurred in the extended LE regimen group and two miscarriages occurred in the conventional LE regimen group. One patient in each group experienced preterm delivery at 33–34 weeks with good neonatal outcomes. Additionally, no early neonatal death or congenital birth defects were detected.

## Discussion

This is the first prospective randomized controlled trial to illustrate the ovarian response, hormone profile, and pregnancy outcomes of patients with PCOS using an extended LE regimen compared with a conventional LE regimen. Our results showed that the rate of ovulation among patients receiving an extended LE regimen was slightly higher than the rate with a conventional LE regimen group. However, this difference did not reach statistical significance. Our findings suggest that it is not necessary to replace the conventional LE regimen with an extended LE regimen in patients with PCOS during their first ovulation induction cycle.

The major concern with regard to extending LE duration was multi-follicular growth. Fouda and Sayed (2011) first documented the use of an extended LE regimen. That study enrolled 106 normal-ovulatory patients treated with LE 2.5 mg per day from cycle days 1 to 9 and revealed a clinical pregnancy rate of 18.96% (40/211) and a multiple pregnancy rate of 4/40 (10%) in those patients (Fouda and Sayed, 2011). Although no cases of OHSS were reported and all the multiple pregnancies were twins, the average number of follicles >18 mm on the day of hCG administration reached 2.24 ± 0.80 after 9 days of LE treatment (Fouda and Sayed, 2011). In our trial, the LE treatment duration was prolonged to 7 instead of 10 days. Although the average number of preovulatory follicles was comparable between treatment groups, one woman using the extended LE regimen yielded four dominant follicles but failed to conceive in this cycle. The most ideal case during ovulation induction therapy would be mono-follicular development and subsequent mono-ovulation and singleton pregnancy (Balen *et al.*, 2016; Birch Petersen *et al.*, 2016; Teede *et al.*, 2023). Although all the conceptions were singletons without neonatal anomalies in our trial, the potential risk of multi-follicular development and multiple pregnancies that would require more monitoring and result in higher costs by applying the extended LE regimen could not be ignored. Therefore, we do not advise to use the extended LE regimen for standard ovulation induction in women with PCOS. Nevertheless, it could be applied as a feasible alternative in a specific population with PCOS who fail to respond to a conventional LE regimen.

Applying an exogenous hCG to trigger ovulation is a popular practice prior to intercourse or intrauterine insemination in women with ovulatory dysfunction (Palatnik *et al.*, 2012; Hancock *et al.*, 2021; Chen *et al.*, 2023; Dai *et al.*, 2023). However, the optimal follicular diameters to apply trigger drugs remain a controversy. Palatnik *et al.* observed a higher pregnancy rate when the leading follicles were triggered at a size of 23–28 mm during intrauterine insemination cycles with LE (Palatnik *et al.*, 2012), while Hancock *et al.* identified hCG administration at a lead follicle size of 21.1–22.0 mm was associated with higher pregnancy rates in patients with ovulatory dysfunction or unexplained infertility during intrauterine insemination treatments (Hancock *et al.*, 2021). From our perspective, it is preferable that dominant follicles rupture spontaneously without the application of exogenous trigger drugs. As a type of aromatase inhibitor, LE could promote FSH production and follicular growth by inhibiting estrogen biosynthesis (Wang *et al.*, 2019; Pundir *et al.*, 2021; Tsiami *et al.*, 2021; Franik *et al.*, 2022; Liu *et al.*, 2023). Owing to the short half-life of LE (∼45 h), serum E_2_ values could rapidly rise after the cessation of LE treatment, and then evoke substantial LH secretion and subsequent ovulation (Rose and Brown, 2020; Yang *et al.*, 2021). All the participants with dominant follicles achieved spontaneous ovulation without exogenous trigger drugs in our trial. The strategy of waiting for the spontaneous LH surge ensured that there was sufficient time for estrogen biosynthesis and endometrial development. As a result, the mean endometrial thickness before ovulation was comparable between the treatment groups, which was in consistent with previous studies using the extended regimens (7–10 days) (Fouda and Sayed, 2011; Zhu and Fu, 2023). In other words, a duration-dependent reduction in endometrial thickness was not observed in our trial. Furthermore, the clinical pregnancy rate and the live birth rate in patients using the extended LE regimen were similar to those in patients with the conventional LE regimen. Therefore, it is not necessary to worry about the reduced endometrial thickness during ovulation induction cycles with the extended LE regimen.

One of the main limitations is that the sample size of our trial was rather small. In addition, one should be cautious when interpreting our findings as the patients enrolled in the present study were lean patients with PCOS with a mean BMI of 23–25 kg/m^2^, and the incidence of patients with LE resistance (∼15%) was less than that reported in most prior articles (Ramezanzadeh *et al.*, 2011; Legro *et al.*, 2014; Wu *et al.*, 2016; Amer *et al.*, 2017; Mejia *et al.*, 2019; Shi *et al.*, 2022; Dai *et al.*, 2023; Sharma *et al.*, 2023), limiting the applicability of our results in obese patients. Nevertheless, we believe that our cohort was a good representation of the Chinese PCOS community as the mean BMI was 22.2 ± 4.2 kg/m^2^ and the prevalence of patients with a BMI≥23 kg/m^2^ was 34.09% (284/833) based on a large community study of Han Chinese patients (Li *et al.*, 2013). Moreover, it may be argued that the definition of PCOS adopted in our trial was outdated because the threshold for polycystic ovary morphology was having more than 20 antral follicles in at least one ovary, according to the recent guidelines published in 2023. However, the Rotterdam criteria remain the most universally accepted diagnostic criteria for PCOS in practice. Besides, there were very few participants with clinical or biochemical hyperandrogenism enrolled in our trial, thus further studies with different PCOS subtypes in a larger population are needed to validate our findings. Additionally, the primary outcome in the present trial was ovulation rate. We acknowledge that the rates of clinical pregnancy or live birth would have been a more suitable parameter for comparing the ovulatory effects during ovulation induction treatments. Nevertheless, the formation of dominant follicles is the first and core step of successful ovulation induction. It may be more meaningful to evaluate the pregnancy rate and delivery rate on the premise of differences in ovulation rate. Another concern was that the treatment allocation was not blinded to the participants and investigators. The combined assessment of ultrasound and serum hormone levels ensured the objectivity and accuracy of the data relevant to ovulation, which may reduce any bias from an open-label design. Finally, cumulative ovulation rates and pregnancy outcomes were not available as only one ovulation induction cycle was incorporated into our study. Additional studies could be conducted to obtain cumulative outcomes in patients with PCOS using an extended LE regimen and provide detailed records relevant to side effects and follow-up of newborns (Legro *et al.*, 2020).

Our data supported the use of a conventional LE regimen in patients with PCOS undergoing their first ovulation induction cycle because a statistically superior effect of the extended LE regimen over a conventional regimen was not detected. Additional prospective trials with larger sample sizes and different subgroups of PCOS are needed to assess the ovulatory effects of different LE treatment durations.

## Data Availability

The data underlying this article will be shared on reasonable requests made to the corresponding author.
